# Molecular determination of genetic diversity among *Campylobacter jejuni* and *Campylobacter coli* isolated from milk, water, and meat samples using enterobacterial repetitive intergenic consensus PCR (ERIC-PCR)

**DOI:** 10.1080/20008686.2020.1830701

**Published:** 2020-10-06

**Authors:** Aboi Igwaran, Anthony Ifeanyi Okoh

**Affiliations:** a SAMRC Microbial Water Quality Monitoring Centre, University of Fort Hare, Alice, South Africa; b Applied and Environmental Microbiology Research Group (AEMREG), Department of Biochemistry and Microbiology, University of Fort Hare, Alice, South Africa

**Keywords:** *Campylobacter*, dendrogram, diversity, cluster, ERIC-PCR, genotyping

## Abstract

Consumption of contaminated meat, milk, and water are among the major routes of human campylobacteriosis. This study aimed to determined the genetic diversity of *C. coli* and *C. jejuni* isolated from meat, milk, and water samples collected from different locations. From the 376 samples (meat = 248, cow milk = 72, and water = 56) collected, a total of 1238 presumptive *Campylobacter* isolates were recovered and the presence of the genus *Campylobacter* were detected in 402 isolates, and from which, 85 and 67 isolates were identified as*C. jejuni* and *C. coli* respectively. Of which, 71 isolates identified as *C. coli* (n = 35) and *C. jejuni* (n = 36) were randomly selected from meat, milk, and water samples and were genotyped using enterobacterial repetitive intergenic consensus PCR (ERIC-PCR). The digital images of the ERIC-PCR genotype were analyzed by GelJ v.2.0 software. The diversity and similarity of the isolates were assessed via an unweighted-pair group method using average linkages clustering algorithm. The results showed that the 36 *C. jejuni* strains separated into 29 ERIC-genotypes and 4 clusters while the 35 *C. coli* were delineated into 29 ERIC-genotypes and 6 clusters. The study revealed the genetic diversity among *C. coli* and *C. jejuni* strains recovered from different matrices characterized by Gelj.

## Introduction

Campylobacters are commensal members of the gut microbiota of livestock, poultry [1], wild birds [2], and are also found in aquatic milieu [3]. Several *Campylobacter* species are known to cause infections and campylobacteriosis are largely caused by *C. coli* and *C. jejuni* [4,5]. Campylobacteriosis cases have been reported in several parts of the world including South America, Africa, North America, Asia, and Europe [6]. Campylobacteriosis is usually associated with consumption of contaminated vegetables, fruits, raw milk, untreated water, foodstuffs, and undercooked meats [4]. About 50%–80% of human campylobacteriosis cases are caused by chicken consumption [7]. Nevertheless, cattle-related campylobacteriosis cases through consumption of unpasteurized milk or undercooked beef have also been reported [8]. *Campylobacter* species including *C. coli* and *C. jejuni* are often isolated and detected in sewage, milk, water, and meats samples [9].

In developing countries, *C. jejuni* and *C. coli* are the two major *Campylobacter* species known as potential etiological agents of acute diarrhea especially in children [10]. *Campylobacter* infections are ecologically diverse [11] and as a result of the burdens and public health impact of *Campylobacter* species, studies on epidemiological analyses, genetic diversity, and similarity of these bacteria species are very significant [4]. Hence, molecular typing techniques are used for the determination of the similarities of isolates and these have shown in better detection of *Campylobacter* strains [12]. Molecular typing techniques with high sensitivity and specificity are recognized as gold standard test for epidemiological investigations of some pathogens including *Campylobacter* species [13,14]. Molecular typing of bacteria isolates is useful to characterize the genetic similarity of the isolates [15] and also helpful in conducting epidemiological studies designed at source tracking of sporadic campylobacteriosis cases by providing information on the genetic *Campylobacter* subtypes in circulation [12]. Molecular genotyping procedures have been used for the detection of intra-species variability of a specific microorganism [16]. Molecular typing methods are assessed base on their performance and ease in usage [17]. Some molecular typing techniques used for genotyping of bacteria isolates include multilocus sequence typing (MLST), gene sequencing-based methods, phylogenetic analysis [18], PCR-ribotyping, PCR sequencing, and enterobacterial repetitive intergenic consensus PCR (ERIC-PCR) [19,20]. ERIC-PCR technique is a simple tool used to differentiate bacteria strains isolated from diverse sources. This technique is a strong tool for the exploration of prokaryotic genomes and has been reported to have improved reproducibility and high discriminatory power [21]. ERIC-PCR is a repetitive element-based PCR technique [22]. The pros of ERIC-PCR over other molecular typing techniques include the capacity to distinguish between closely related bacteria strains as well as being quick, simple, cheap, dependable, and high-throughput genotyping method [23]. Molecular typing of *Campylobacter* species by ERIC-PCR has been reported to show high discriminatory power in strains diversity [24], and the explanation of banding patterns of ERIC-PCR DNA fingerprinting by visual judgment is an onerous task particularly when relating to multiple band patterns, distant, and diverse bands. However, GelJ is a tool that simplify this task and is used in analyzing DNA fingerprint gel images to overcome these limitations [25]. In South Africa, there is paucity of information on genotyping of *C. coli* and *C. jejuni* isolated from milk, water and meat samples and there is no report on molecular genotyping of *Campylobacter* species in the study area. Since it is not possible to determine the genetic relatedness of isolates using ordinary PCR approach; therefore, the study evaluated the genetic diversity of *C. jejuni* and *C. coli* isolated from water, milk, and meat samples using ERIC-PCR techniques.

## Material and methods

### Sources of bacteria strains

A total of 376 samples (Meat = 248, cow milk = 72, and water = 56) were collected in Chris Hani and Amathole District Municipalities, South Africa between March 2019–August 2019. The meat samples [beef, turkey, pork, mutton, and chicken) were obtained from supermarkets, retail shops, and butcheries, the milk samples were obtained from farms, cars/roads side, retail shops, and butcheries while the water samples were obtained from rivers and pond used for irrigation. The meat samples were aseptically packed into different sterile plastic bags while the water and milk samples were collected in sterile 1 L and 250 mL polypropylene bottles, transported to the laboratory for examination in a cooler box with ice packs within six hours of collection. For the microbiological analysis of the samples, the methods described by [26 27], was adopted for isolation of *Campylobacter* from water, milk, and meat samples, respectively, incubated under microaerophilic conditions in 10% CO_2_ in HF151UV incubator for 48 h at 42°C.

### Extraction of bacterial DNA

DNA was extracted by boiling method following the procedure described by [28], with slight modification as reported by [29].

### *Molecular confirmation and identification of* C. jejuni *and* C. coli

Molecular identification of the genus *Campylobacter* was carried out by PCR assay using the primer sets listed in supplementary Table 1 as reported by [30], targeting part of the 16S rRNA gene at 439 bp. The identified genus *Campylobacter* were further delineated into *C. coli* and *C. jejuni* using the primer sets reported by [31], targeting *asK* and *cj0414* genes, respectively, as shown in Table 1.

**Table 1.** Primers sets used for the identification of *Campylobacter* species.

Target strain	Primer sets	Base pairs	Targeted genes	PCR condition	Cycles	Reference
Genus *Campylobacter*	F: GGTGTAGGATGAGACTATATA R: TTCCATCTGCCTCTCCC	439 bp	16S rRNA	95°C for 5 min, followed by 94°C for I min, 58°C for 1 min, 72°C for 2 min, and the final extension was set at 72°C for 2 min.	33	[30]
*C. coli*	F: GCTTCGCATAGCTAACAT R: GGTATGATTTCTACAAAGCGAG	502 bp	*asK*	95°C for 15 min, 95°C for 30 sec, 50°C for 90 min, 72°C for I min and 72°C for 7 min.	25	[31]
*C. jejuni*	F: CAAATAAAGTTAGAGGTAGAATGT R: CCATAAGCACTAGCTAGCTGAT	161 bp	*cj0414*	95°C for 15 min, 95°C for 30 sec, 50°C for 90 min, 72°C for I min and 72°C for 7 min	25	[31]

### *ERIC-PCR typing of* C. jejuni *and* C. coli

ERIC-PCR is a molecular-based method that has been well applied for the discrimination of *Campylobacter* species [32]. From the identified isolates detected as *C. coli* and *C. jejuni*, 71 strains [*C. coli *= 35 and *C. jejuni *= 36) were randomly selected from different sources and the identified isolates were subjected to PCR using the ERIC primer sets R1: ATGAAGCTCCTGGGGATTCAC and R2: AAGTAAGTGACTGGGGTGAGCG following the method described by [33]. The PCR reactions were verified by resolving them in 3% agarose gel in a 5x TBE buffer, stained with ethidium bromide at 90 volts for 240 min and viewed as stated before.

### Clustering analysis and determination of discriminatory power (D]

The DNA fingerprints obtained from the ERIC-PCR technique were analyzed with computer-assisted pattern analysis using the GelJ v.2.0. software [25]. The relatedness of the isolates was compared and dendrograms were constructed by UPGMA and cluster analysis were used to determine the relationships between each isolate. The value of discriminatory power [D) was determined using online calculator for discriminatory power as reported by [34].

### Determination of reproducibility of ERIC-PCR

The capacity of a technique to give the same result when repeated tests are carried out on the same isolate is recognized as reproducibility [22]. The ERIC-PCR analysis of the DNA fingerprints result can be directly correlated within a single PCR experiment. However, to determine the reproducibility of these techniques, the clustered result of the 35 *C. coli* and 36 *C. jejuni* isolates were repeated twice in further ERIC-PCR assay.

## Results

### *Incidence of* Campylobacter *and* Campylobacter *species in the matrices*

**Figure 1.**
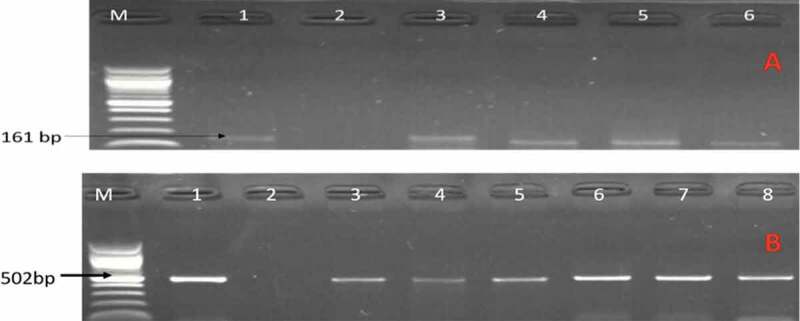
A gel image of PCR confirmed *C. jejuni*. Lane M: 100 bp DNA ladder, lane 1: positive control (*C. jejuni* ATCC 33560), lane 2: negative control, lane 3–6: positive *C. jejuni* isolates (161 bp) while image B are some confirmed *C. coli*. Lane 1: positive control (*C. coli* ATCC 33559), lane 2: negative control, lane 3–8: positive *C. coli* isolates (502 bp).

From culture, a total of 1238 presumptive *Campylobacter* isolates were recovered and the presence of the genus *Campylobacter* was detected in 402 isolates, and from which 85 and 67 isolates were detected to be *C. jejuni* and *C. coli* respectively. Of which 71 isolates identified as *C. coli* (n = 35) and *C. jejuni* (n = 36) were randomly selected from meat, milk, and water samples. Of the 71 identified selected *C. jejuni* and *C. coli* fingerprint, 23 (65.71%) *C. coli* isolates were from meat samples (chicken = 4, pork = 6, beef offals = 13), 11 (31.43%) *C. coli* from milk samples, 1 (2.88%) *C. coli* from water samples, 23 (63.89%) *C. jejuni* were from water samples, 13 (36.11%) *C. jejuni* from meat samples (chicken = 9, beef = 2, and beef offals = 2). Figure 1 shows a gel image of some of the PCR identified *C. coli* and *C. jejuni* isolates.

### *Genetic diversity of* C. jejuni *and* C. coli *isolates*

**Figure 2.**
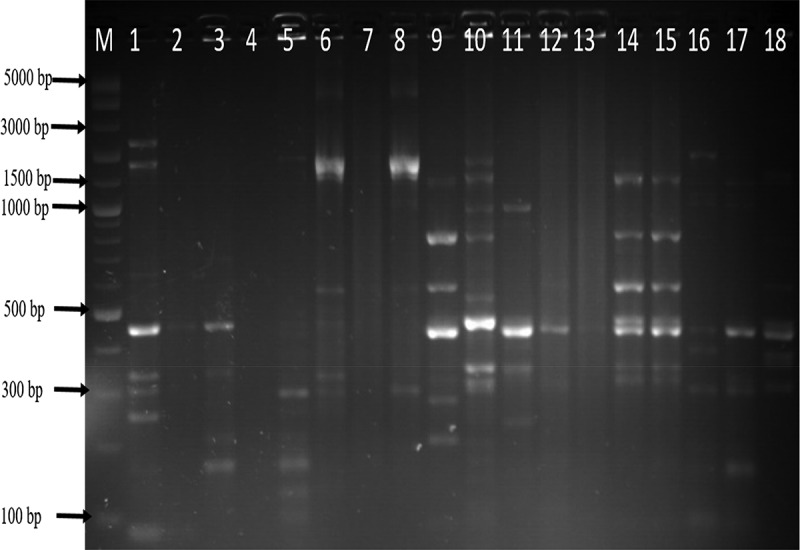
Gel ERIC-PCR amplification results of *C. jejuni* isolates. M, DNA ladder, 1–18: some amplified *C. jejuni* isolates.

**Figure 3.**
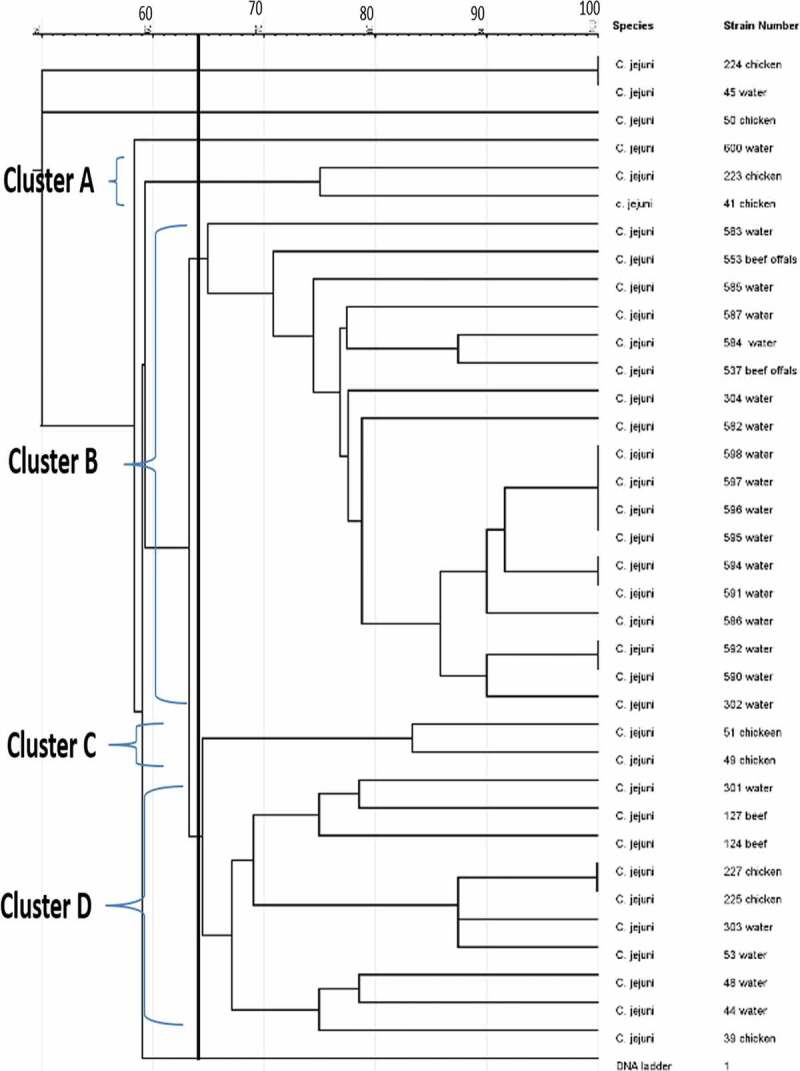
UPGMA dendrogram image obtained from cluster analysis showing the relationship and diversity of 36 *C. jejuni* isolates from different sources using ERIC-PCR technique.

**Figure 4.**
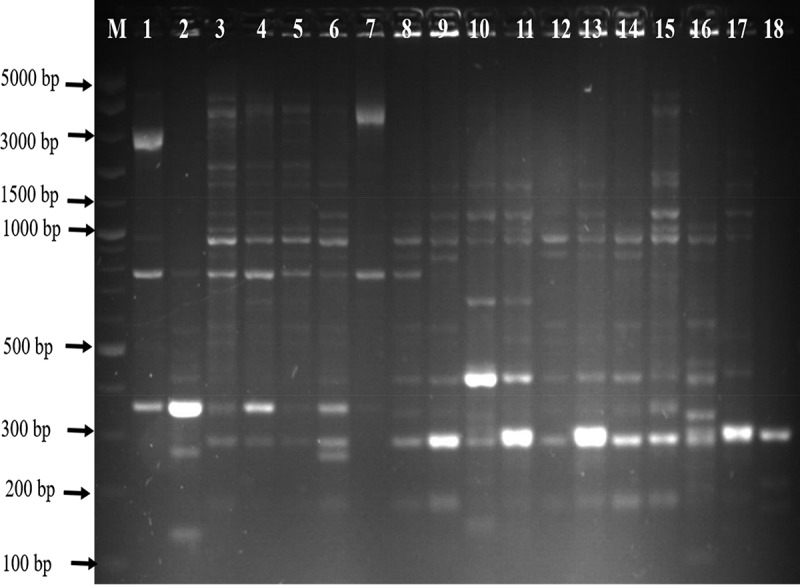
Gel ERIC-PCR amplification results of *C. coli* isolates. M, DNA ladder, 1–18: some amplified *C. coli* isolates.

The genetic fingerprints of *C. jejuni* and *C. coli* from meat, water, and milk were characterized by ERIC-PCR and the dendrogram images obtained from the analysis of ERIC-PCR results were constructed using the GelJ v.2.0. software to define the genetic similarity of the isolates. The ERIC-PCR condition that was previously developed and reported for fingerprints analysis of *Campylobacter* species was used to analyze 71 *Campylobacter* isolates identified as *C. jejuni* (36) and *C. coli* (35). The gel image band patterns of *C. jejuni* and *C. coli* strains varied in relation of the distribution of polymorphic bands ranging from 100 to 4500 bp (Figures 2 and 4). The variances among the two *Campylobacter* species analyze were evaluated on the basis of the migration arrangements of the amplified bands. From the 36 *C. jejuni* isolates from water and meat samples genotyped using ERIC-PCR assay, 35 isolates out of the 36 isolates produced 1–14 bands and the UPGMA dendrogram clustering image separated all the 36 isolates into 29 ERIC-genotypes and the 29 ERIC-genotypic profiles generated were grouped into four clusters at a similarity cutoff of 65% (Figure 3).

**Figure 5.**
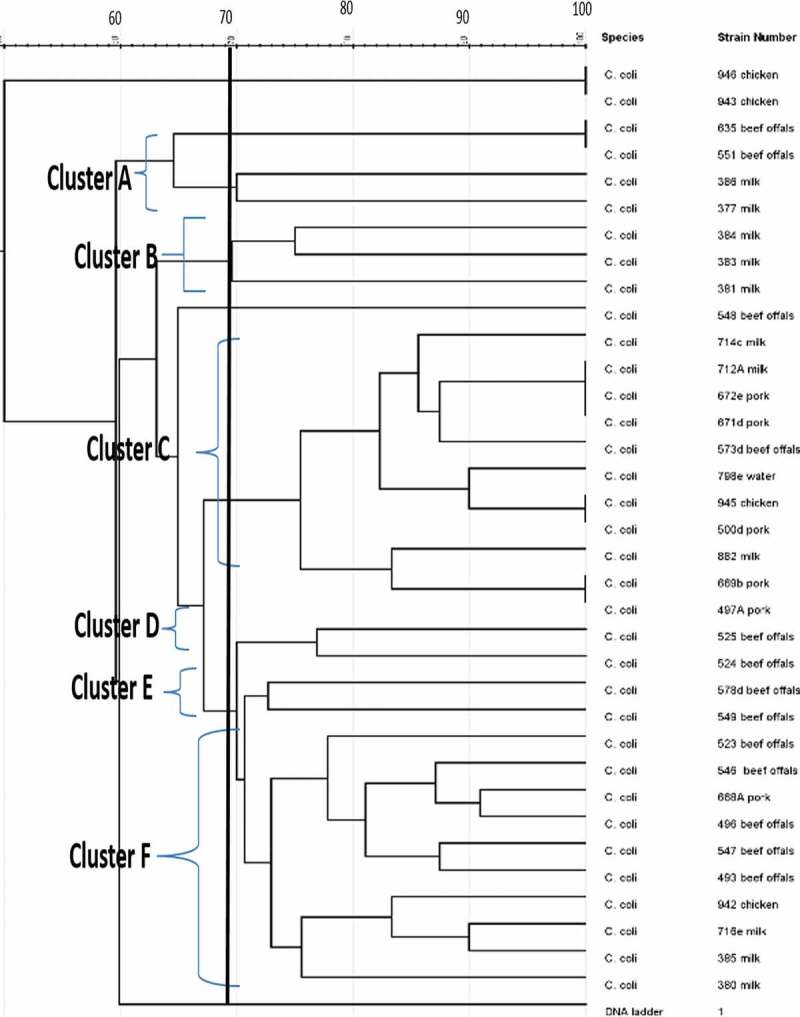
UPGMA dendrogram image obtained from cluster analysis showing the relationship and diversity of 35 *C. coli* isolates from different sources using ERIC-PCR technique.

The dendrogram image obtained from GelJ cluster analysis of *C. jejuni*, the highest ERIC-genotype cluster of *C. jejuni* profiles generated was found in cluster B (composed of 18 isolates) followed by cluster D (composed of 10 isolates) and cluster A and C (composed of 2 isolates each). Some isolates had no polymorphic band and were not group into any cluster. Also, some of the *C. jejuni* strains showed a high degree of relatedness with similarity indices of 100%. Furthermore, the dendrogram image constructed showed that some of the *C. jejuni* isolates recovered from chicken, beef and water were clustered into the same group and this indicates high genetic relatedness among the isolates. Likewise, the ERIC-PCR genotyping result of 35 *C. coli* isolates produced 1–10 bands (Figure 4), and the dendrogram clustering image constructed separated the 35 *C. coli* isolates into 29 ERIC-genotypic profiles at a similarity cutoff of 70%. Furthermore, the dendrogram image shown an important intra-species diversity of *C. coli* strains irrespective of the isolates origins. The 29 ERIC-genotypes generated were grouped into six clusters (Figure 5) and among the clusters, the most prevalent genotypes were in cluster F represented by 10 isolates, followed by cluster C composed of 11 isolates, cluster A composed of 4 isolates, cluster B composed of 3 isolates and Cluster D and E composed of 2 isolates each. The six clusters of *C. coli* generated were visibly separated and in cluster C, *C. coli* isolate recovered from pork and milk showed a high level of genetic relatedness (100%). Similarly, the DNA fingerprints of some isolates obtained from water samples were clustered into the same group with those isolated from meat and milk samples obtained from different locations while some isolates had no band and were not clustered into any group with others.

### Reproducibility

Reproducibility of the experiments was confirmed by the UPGMA clustering results of the 35 (*C. coli*) and 36 (*C. jejuni*) isolates that had clustered together on preceding ERIC-PCR assay, which were observed to continuing to cluster on the following ERIC-PCR assay.

### Discussion

Molecular typing techniques including ERIC-PCR have been revealed to shown high genomic diversity of *Campylobacter* species, reflecting its quick adaptive changes during infection [21]. Some *Campylobacter* species are well known to cause infections and it is vital that *Campylobacter* species are correctly differentiated into intra-species diversity. The present study revealed the genetic diversity of *C. coli* and *C. jejuni* obtained from different sources using ERIC-PCR DNA fingerprinting assay. DNA fingerprinting assay is used for comparing DNA patterns, which allows the analysis of the genomic similarity among different samples and categorizes them into clusters [25]. From the PCR results, a total of 71 isolates identified as *C. jejuni* and *C. coli* from milk, water, and meats were randomly selected and genotyped by ERIC-PCR. ERIC-PCR study has been used to amplify different DNA regions to generate genetic patterns that are precise for specific isolates [35]. The result from the ERIC-PCR fingerprinting of both *C. coli* and *C. jejuni* clustered the isolates into six and four clusters, respectively, and this result suggests that the isolates from meats, milk, and water sources have high genetic diversity.

Though clustering of *C. jejuni* and *C. coli* isolates from different sampling sites and source propose evolutionary relatedness of the isolates. This result revealed that investigation on genotyping of different *C. jejuni* and *C. coli* strains using ERIC-PCR showed better detection of the diverse strains and these results is in accordance with the reported of [36–38]. Furthermore, *C. coli* and *C. jejuni* isolates obtained from different matrices were grouped either in the same or different clusters. The ERIC-PCR analysis separated distinguishingly the 36 *C. jejuni* isolates from water and meat samples into 34 ERIC-genotypes and this indicates genetic diversity among *C. jejuni* isolates recovered from the two sources. This result is in akin with the reports of [39–41], who have also reported high diversity of *Campylobacter* species obtained from porcine, avian, turkey, and broiler, respectively. The UPGMA dendrogram clustering image of the 36 *C. jejuni* isolates genotyped grouped the isolates into four clusters and this finding is in line with the report of [42,43]. Result obtained from *C. jejuni* fingerprinting is contrary to the report of [44], who in their report revealed that 15 *C. jejuni* genotyped were grouped into 10 genotypes with 3 clusters. *C. jejuni* have a natural capacity for gene rearrangements and gene transfer in the genome which might likely explain the increase in its genomic heterogeneity [45]. The UPGMA dendrogram image generated from the genotyping analysis of *C. jejuni* isolates revealed high level of genetic diversity among *C. jejuni* isolates and our result corresponds with the report of [46]. The dendrogram image of *C. jejuni* isolates showed that some isolates were highly diverse while some were 100% genetically related and this shows the genetic multiplicity of *C. jejuni* strains analyzed. This result corresponds with the report of [47], who also revealed the genetic multiplicity of *C. jejuni* isolates recovered from aquatic sources. In the report of [48], multiple strains of *C. jejuni* belonging to four distinct clades were revealed to be implicated in human campylobacteriosis outbreak as a result of the consumption of undercooked chicken liver pâte. Multiple strains or co-infection of human campylobacteriosis cases involving more than one *C. jejuni* subtype might occur in up to 10% of most *Campylobacter* infection cases [49].

In the study conducted by [50], multiple *C. jejuni* subtypes were detected in chickens. In addition, some of the *C. jejuni* subtypes causing human infection may be minor strains in the chicken microflora [48]. Furthermore, study has also shown that *C. jejuni* has caused the highest zoonosis menace to public health compared to other *Campylobacter* species [51]. Another *Campylobacter* species reported to cause high rate of campylobacteriosis is *C. coli* and the ERIC-PCR profile of *C. coli* genotyped showed that some of the *C. coli* strains were genetically diverse, demonstrating the occurrence of various genotypes of *C. coli* in the study area. It was also observed that some *C. coli* strains had no polymorphic band while some showed a high level of genetic similarity of 100%. Furthermore, the 35 *C. coli* isolates analyzed were grouped into 29 genotypes and this result is similar with the report of [52], who in their study obtained 22 genotypes from 65 *C. coli* isolates analyzed. Also, the dendrogram revealed the genetic diversity among the *C. coli* isolates recovered from the same or different sources. Simultaneously, high genetic relatedness was also observed among some *C. coli* isolates recovered from milk and pork as seen in cluster C in Figure 5 and this result corroborates with the report of [8]. The finding also revealed that *C. coli* isolate recovered from aquatic milieu was closely related with those from other sources and this result corresponds with the report of [53]. Detection of genotypic relatedness among some *C. coli* and *C. jejuni* isolates recovered from aquatic milieu and meat sources confirmed that livestock and poultry are sources of environmental spread of *Campylobacter* species and these results is in accordance with the report of [54]. Genetic diversity is one of the different mechanisms that helps pathogens to thrive in unfriendly circumstances within the environment or in the host given them the ability to colonize multiple hosts [55]. Genomic rearrangement and horizontal gene transfer are among the diverse phenomena that can cause genetic diversity in *C. jejuni* and *C. coli* [45]. The observed various band patterns suggest the presence of genetically diverse strains of *C. coli* and *C. jejuni* analyzed.

This method used in *Campylobacter* genotyping is very sensitive in identifying or detecting variances between isolates of the same species and also provide discerning way for discriminating *C. coli* and *C. jejuni* strains and this result is in line with the report of [56]. In this study, the ERIC-PCR result gave a discriminatory power [D) value of 0.78 for *C. coli* and 0.60 for *C. jejuni* and this is a good value and the result is akin with the report of [46]. This is the first report from the study area on the application of ERIC-PCR for genotyping of *Campylobacter* species from different sources. The DNA profiles were visibly noticeable through precise fingerprint arrangements. The ERIC-PCR is therefore suggested to be used as simple and cheap tool for the determination of diverse strains of bacterial species. The primer sets used in this study aided in differentiating closely related strains within the same *Campylobacter* species and the results from this study revealed a wide genomic multiplicity of *C. jejuni* and *C. coli* recovered from water, milk and meat samples. However, the isolates analyzed in this present study are not representative of the *Campylobacter* populace and we are guarded that no generalization is deduced from the study. More investigation would provide more data on the epidemiology of *Campylobacter* in the study area.
